# Reference intervals for blood-based biochemical analytes of southern Beaufort Sea polar bears

**DOI:** 10.1093/conphys/coz040

**Published:** 2019-09-16

**Authors:** Tricia L Fry, Kristen R Friedrichs, Todd C Atwood, Colleen Duncan, Kristin Simac, Tony Goldberg

**Affiliations:** 1 Pathobiological Sciences, School of Veterinary Medicine, University of Wisconsin, Madison, USA; 2 Alaska Science Center, US Geological Survey, Anchorage, AK, USA; 3 Department of Microbiology, Immunology and Pathology, College of Veterinary Medicine and Biomedical Sciences, Colorado State University, Fort Collins, CO, USA

**Keywords:** Arctic, blood biochemistry, polar bear, reference interval, serum analytes, wildlife health

## Abstract

Accurate reference intervals (RIs) for commonly measured blood-based analytes are essential for health monitoring programmes. Baseline values for a panel of analytes can be used to monitor physiologic and pathophysiologic processes such as organ function, electrolyte balance and protein catabolism. Our reference population includes 651 serum samples from polar bears (*Ursus maritimus*) from the southern Beaufort Sea (SB) subpopulation sampled in Alaska, USA, between 1983 and 2016. To establish RI for 13 biochemical analytes, we defined specific criteria for characterizing the reference population and relevant subgroups. To account for differences in seasonal life history characteristics, we determined separate RI for the spring and fall seasons, when prey availability and energetic requirements of bears differ. We established RI for five subgroups in spring based on sex, age class and denning status, and three subgroups in fall based on sex and age class in females only. Alkaline phosphatase activities were twice as high in subadult as in adult polar bears in spring (*z*_males_ = 4.08, *P*_males_ < 0.001, *z*_females_ = 3.90, *P*_females_ < 0.001) and did not differ between seasons. Denning females had significantly higher glucose concentrations than non-denning females (*z* = 4.94, *P* < 0.001), possibly reflecting differences in energy expenditure during lactation. A total of 10 of the 13 analytes differed significantly between seasons in either males or females; however, the physiologic importance of these differences may be minimal. Establishing these RIs allows for temporal monitoring of polar bear health in the SB and may prove useful for assessing and monitoring additional polar bear subpopulations in a changing Arctic environment.

## Teaser

Reference intervals (RIs) for biochemical analytes provide baseline values for assessing physiologic status and health. We established RIs for commonly assessed blood-based analytes for polar bears from the southern Beaufort Sea. These RIs fill a knowledge gap that will inform assessments of polar bear health in a changing Arctic.

## Introduction

Climate change is rapidly affecting the Arctic region. Arctic ocean temperatures have risen at over twice the average rate of global warming with models suggesting that the Beaufort Sea could increase 4°C above the 1981–2010 average by 2040 (Overland *et al.*, 2018), accelerating abiotic and biotic changes (IPCC, 2018). With warming temperatures and changes in sea ice phenology, polar bears (*Ursus maritimus*) are being exposed to novel stressors related to changes in habitat, nutrition, competition, and pollutants (Burek *et al.*, 2008). Observed effects associated with environmental changes in polar bears include increased rates of fasting (Cherry *et al.*, 2009; Rode *et al.*, 2018), declines in body condition and cub recruitment, (Rode *et al.*, 2010, 2012, 2014; Obbard *et al.*, 2016) and declines in survival and abundance (Regehr *et al.*, 2007; Bromaghin *et al.*, 2015; Obbard *et al.*, 2018). However, the effects of chronic environmental stressors on metabolic processes, physiologic function and health are poorly understood (Atwood *et al.*, 2015; Bowen *et al.*, 2015; Fagre *et al.*, 2015; Patyk *et al.*, 2015). Thus, there is a critical need to describe biomarkers that can be used as a component in monitoring polar bear health (Friedrichs, 2009; Patyk *et al.*, 2015).

A common method for assessing physiologic function and pathology in animals is to measure blood-based analytes, which include measures of organ system function, electrolytic balance, enzyme activity, protein abundance and nutrition. Deviations from expected values of blood-based analytes are commonly used to ascertain pathologic states (Friedrichs *et al.*, 2012). A precursor to effectively using such indices is establishment of reference intervals (RIs), which are baseline values for each analyte derived from a normal, healthy reference population. Grasbeck and Saris (1969) first introduced the concept of theoretical RI as values obtained under controlled conditions with `healthy, normal’ individuals as the reference population (Grasbeck, 1990). A RI is mostly commonly delimited by the central 95% of the reference population with the low and high limits bounding the interval (Geffré *et al.*, 2009; Friedrichs *et al.*, 2012). Hanks (1981) outlined the usefulness of blood-based variables to assess physical condition and health status of wildlife as well as to assess disease status and changes in the environment. Friedrichs (2009) further suggested that RI could be used to assess the physiologic health of individuals, populations or ecosystems.

When calculating RI, it is important to consider life history variables that may influence blood-based analytes of individuals. In wild, free-living animals, this may include seasonal impacts. Seasonal fluctuations in blood biochemistry can result from a variety of factors, including diet and nutrition, reproduction, behaviour and metabolic requirements (Lathi, 2004; Friedrichs *et al.*, 2012). Ursids demonstrate substantial variation in biochemical values depending on habitat, behaviour and diet (Lee *et al.*, 1977; Matula *et al.*, 1980; Nelson *et al.*, 1983; Brannon, 1985; Schroeder, 1987; Franzmann and Schwartz, 1988; Ramsay *et al.*, 1991; Tryland *et al.*, 2002). Identifying seasonal changes in RI is especially important for polar bears, given the extreme seasonality of their life history and physiologic adaptations, such as hyperphagia in the spring and extended fasts in other seasons (Atkinson and Ramsay 1995; Cherry *et al.*, 2009; Rode *et al.*, 2018).

Our objective was to use the southern Beaufort Sea (SB) subpopulation of polar bears to define RI that can be used to monitor the health of the SB subpopulation and for comparisons to other subpopulations. Specifically, we used polar bear blood chemistry values collected over 34 years (1983–2016) to define RI for 13 common serum analytes that measure liver and kidney function and status, immune system activity, dietary intake and electrolyte and mineral balance. We also examined variation in analytes across subgroups, such as denning status, age and sex, in both spring and fall.

## Materials and methods

**Figure 1:**
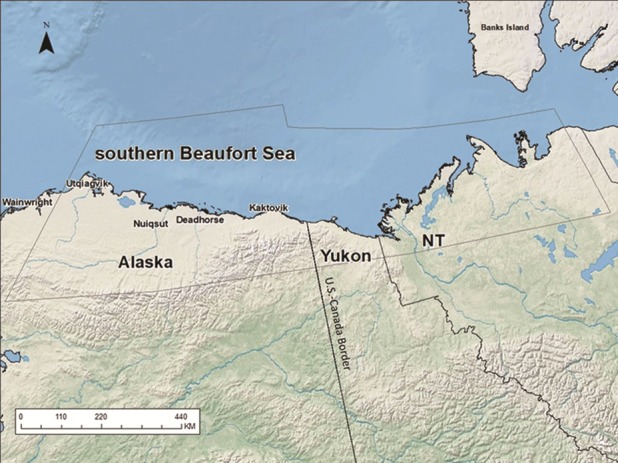
Between 1983 and 2016, polar bears were captured within the International Union for Conservation of Nature defined boundary (thin line) for the SB subpopulation between Icy Cape, Alaska, and the United States–Canada Border.

Polar bears were captured, sampled and released on the sea ice of the SB, Alaska, as part of a long-term research programme. Spring captures most commonly occurred on sea ice from 1983 to 2016 typically between March 20th and May 5th. Fall captures took place on sea ice and on land between August and November, intermittently from 1983 to 2009. The study area included the Alaska portion of the SB subpopulation, bounded by Icy Cape, Alaska, to the west and the United States–Canada border on the east and extended from the coast to ~90 km over sea ice in most years (Fig. 1). Polar bears were located from a helicopter and immobilized with a rapid-injection dart (Palmer Cap-Chur Equipment, Douglasville, Georgia, USA) containing Sernylan or M-99 prior to 1987 and thereafter, zolazepam-tiletamine (Telazol^®^ or Zoletil^®^; Stirling *et al.*, 1989). Immobilized bears were aged, weighed to the nearest kg and marked with an ear tag number and a unique tattoo on the upper lip. Polar bears ≥5 years old were classed as adults, and 3- and 4-year-old polar bears were classed as subadults. Denning status was ascertained when a female polar bear was captured with young of the year. Capture and handling of polar bears were conducted under appropriate research permits, including Marine Mammal Research Permit MA690038-17.

We collected blood into evacuated plain tubes (Vacutainer; BD Biosciences, Franklin Lanes, NJ) by venipuncture of the femoral vein. Whole blood was stored in a cooler with chemical heat packs to prevent freezing until returning from the field, at which point serum was separated from blood by centrifugation at 1500 g for 5 min (TRIAC; Clay Adams, Parsippany, NJ) and frozen at −20°C. At the conclusion of the field season, sera were stored at −70°C until analysed. Sera were analysed using a VetScan VS2 Biochemistry Analyser (Abaxis, Union City, CA) to measure the following analytes: alanine aminotransferase (ALT), alkaline phosphatase (ALP), albumin (ALB), blood urea nitrogen (BUN), calcium (CA), creatinine (CREA), glucose (GLU), phosphorus (PHOS), potassium (POT), sodium (NA), total bilirubin (TBIL) and total protein (TP). Globulin (GLOB) was calculated by subtracting ALB from TP. These analytes comprise the comprehensive diagnostic profile defined by Abaxis. The functional and interpretive characteristics of each analyte are summarized in Table 1 (Stockham and Scott, 2013). We established RI based on the guidelines of the American Society of Veterinary Clinical Pathology (Friedrichs *et al.*, 2012). We calculated RI for each of the 13 serum analytes using the Excel macros Reference Value Advisor (Geffré *et al.*, 2011). Outliers were removed based on Dixon’s range statistic (see Geffré *et al.*, 2011). In addition, individuals with two or more outliers in their analyte panel were excluded from the reference population under the assumption that this may indicate a deviation from health.

**Table 1:** Summary of blood-based analytes

Analyte	Tissue source or function^a^	Brief interpretive use
ALT	Liver and muscle	Increases in some hepatic and severe muscle disorders
ALB	Synthesized by liver, source of amino acids, acts as carrier protein	Increase with dehydration; decreases in some liver, renal, and inflammatory disorders
ALP	Primarily liver and bone	Increases in some liver and bone disorders, increases during active bone growth (juveniles)
TBIL	Product of erythrocyte catabolism, processed by the liver and eliminated in bile	Increases with hemolysis or in disease of the liver and biliary system
BUN	Product of protein catabolism, source of nitrogen for protein synthesis, eliminated primarily by kidney	Decreases with low protein intake and liver failure, increases with high protein meals and with decreased renal elimination (↓GFR)
CA	Structural component of bone; important cation for enzymatic, neurologic and muscular function	Approximately 50% bound to ALB, may be altered by vitamin D disorders
PHOS	Structural component of bone, important anion for energy generation (ATP)	Increases with decreased renal elimination (↓GFR)
CREA	Catabolic product of muscle, eliminated through kidney	Low muscle mass results in lower basal concentrations, increases with decreased renal elimination (↓GFR)
GLU	Energy metabolite derived from food intake and hepatic synthesis, stored as glycogen in the liver	Strictly regulated by insulin, glucagon and other hormones, increased by glucocorticoid secretion (termed a stress response)
NA	Important cation for osmoregulation	Strictly regulated by several hormonal systems and renal function
POT	Important cation for neurologic and muscular activity	Strictly regulated by several hormonal systems and renal function
TP	Comprised of ALB and many different GLOB molecules	Changes in TP are reflected by changes in ALB, GLOBs or both
GLOB	Comprised of many different protein molecules that function in immunity and coagulation and as carrier molecules	Increased GLOBs indicate an immune response of significant duration (several days or more), individual GLOBs can be measured for specific information

GFR means glomerular filtration rate, a measure of kidney function; GFR decreases with dehydration, renal failure and urinary bladder obstruction

a ^a^Functional and interpretive characteristics are described by Stockham and Scott (2013).

We defined subgroups based on age class, sex and denning status, each of which may influence physiologic processes (Friedrichs *et al.*, 2012) as well as samples size. To reflect the life history traits of polar bears, spring RIs were calculated for five subgroups (females: non-denning adults, denning adults and subadults; males: adults and subadults (Table 2), and fall RIs were calculated for three subgroups (female adults, female subadults and males). Males were not further subdivided by age class in fall in order to maintain a sample size ≥20 (Friedrichs *et al.*, 2012). The decision to create only one subgroup for males in fall was strengthen because none of the analytes had a difference in means >25% (Sinton *et al.*, 1986), and confidence intervals between the two age groups overlap for all analytes with the exception of BUN. All samples were independent; an individual polar bear was only in a subgroup once.

**Table 2:** Number of polar bears sampled by season

	Spring		Fall	
	Females	Males	Females	Males
Adults (non-denning)	184	161	114	18
Subadult	43	30	38	15
Denning adults	48	−	−	−

RIs were calculated using non-parametric methods when samples sizes were adequate (*n* ≥ 40). We used parametric analyses when 20 < *n* < 40 and the distribution was Gaussian. We used a BoxCox transformation with parametric analysis when transformation to a Gaussian distribution was necessary (Daly *et al.*, 2017). Upper and lower confidence intervals were calculated using non-parametric bootstrap methods when 20 ≤ *n* ≤ 120 and according to tables when 120 ≤ *n* ≤ 370 (CLSI 2008; Geffré *et al.*, 2011). When data could not be transformed to a Gaussian distribution, RIs were defined as the minimum and maximum values with lower and upper 90% confidence intervals excluded. To assess statistical differences between subgroups and season, we compared the means of each analyte using a generalized linear model with Tukey’s multiple comparison of the means. We assessed physiologic importance of differences in RI using the upper and lower confidence intervals between subgroups and seasons; if the upper or lower reference limit was bounded by the comparative subgroup confidence interval, the RIs were considered to have limited physiologic difference.

## Results

Our reference population included 651 polar bear samples (Table 2). Bears in the reference population had a body condition score ≥3 (ranking 1–5, with 5 = obese; Stirling *et al.*, 2008) and had unremarkable physical exams. A summary of RIs, including sample size, summary statistics and 90% upper and lower confidence intervals for each of the 13 analytes, is reported in Table 3 for female polar bears and Table 4 for male polar bears as well as statistically significant differences between subgroups. We report both statistical and physiologic differences in our results. Outliers were identified in the analysis of 32 out of 104 RIs. In 20 RI calculations, the outliers represented < 9% of the reference population, and in cases where outliers represented a greater percentage of the reference population, the sample size was small (*n* < 8). Outliers were distributed throughout the duration of the study.

**Table 3:** RIs for female polar bears from the SB

Analytes	Units	Season	Subgroup	*n*	Mean	Standard deviation	Median	Minimum	Maximum	RI	90% Confidence interval for lower limit	90% Confidence interval for upper limit
ALT	U/l	Spring	Adult^*^^,^^a^	162	50	25	43	11	160	16–126	11–25	96–160
			Denning^a^	32	33	9	35	13	55	14–53	9–18	48–58
			Subadult^c^	33	27	9	25	14	48	13–49	11–15	42–58
		Fall	Adult	110	28	12	27	11	70	12–65	11–13	50–70
			Subadult	37	27	8	26	15	43	14–45	12–16	40–51
ALB	g/dl	Spring	Adult^a^	179	5.5	0.4	5.6	4.4	6.4	4.7–6.3	4.4–4.9	6.2–6.4
			Denning^c^	48	5.3	0.4	5.2	4.3	6.0	4.4–6	4.4–4.7	5.9–6
			Subadult^a,c^	42	5.5	0.4	5.4	4.7	6.2	4.7–6.2	4.7–5.1	6–6.2
		Fall	Adult	111	5.5	0.5	5.6	4.2	6.5	4.3–6.5	4.2–4.7	6.3–6.5
			Subadult	38	5.6	0.3	5.6	5.0	6.2	4.9–6.3	4.8–5.1	6.1–6.4
ALP	U/l	Spring	Adult	165	40	27	32	6	139	8–116	6–10	91–139
			Denning	45	32	29	20	5	126	5–122	5–8	94–126
			Subadult^a^	38	66	26	68	26	132	12–120	1–24	108–132
		Fall	Adult^a^	106	32	25	24	3	127	6–115	3–9	88–127
			Subadult	36	64	41	50	10	173	11–181	7–18	141–229
BUN	mg/dl	Spring	Adult	187	16.7	13.1	12.0	2.0	57.0	2.4–48.8	2–2.7	45–57
			Denning	49	15.0	12.3	11.0	1.0	47.3	1.4–46	1–3	39.5–47.3
			Subadult	42	16.6	14.2	11.8	1.0	53.5	1–53.5	1–3	44–53.5
		Fall	Adult	113	16.6	18.0	9.5	2.7	114.5	3–70.3	2.7–3.9	53.2–114.5
			Subadult	32	10.2	5.9	8.0	4.0	24.3	4.1–28.9	3.9–4.6	20.2–38.3
CA	mg/dl	Spring	Adult	176	9.9	0.7	9.9	7.5	12.5	8–11.5	7.5–8.8	11.1–12.5
			Denning	48	9.8	0.7	9.8	7.8	12.4	7.8–12	7.8–8.8	10.7–12.4
			Subadult	42	10.2	0.4	10.1	9.4	11.1	9.5–11	9.5–9.6	10.7–11.1
		Fall	Adult	109	10.1	0.9	10.2	7.3	12.7	7.8–11.9	7.3–8.2	11.5–12.7
			Subadult	37	10.4	0.6	10.5	9.3	11.7	9.3–11.6	9.1–9.6	11.3–11.9
CREA	mg/dl	Spring	Adult	187	0.9	0.2	0.9	0.4	1.7	0.6–1.5	0.4–0.6	1.4–1.7
			Denning^a^	49	1.1	0.3	1.0	0.4	1.8	0.5–1.8	0.5–0.7	1.6–1.8
			Subadult	42	0.9	0.2	0.9	0.5	1.4	0.5–1.4	0.5-0.6	1.2–1.4
		Fall	Adult	111	1.0	0.3	1.1	0.4	1.8	0.6–1.7	0.4–0.6	1.5–1.8
			Subadult	38	1.0	0.3	1.0	0.6	1.9	0.6–1.8	0.6–0.7	1.5–2.1
GLOB	g/dl	Spring	Adult	182	1.4	0.4	1.4	0.5	2.7	0.8–2.4	0.5–0.9	2.3–2.7
			Denning	48	1.3	0.5	1.3	0.2	2.3	0.3–2.3	0.2–0.8	2.2–2.3
			Subadult	41	1.3	0.3	1.2	0.8	1.9	0.8–1.9	0.8–1	1.8–1.9
		Fall	Adult^a^	109	2.0	0.7	1.9	0.6	4.4	0.8–3.8	0.6–1.1	3.2–4.4
			Subadult	38	1.7	0.3	1.6	1.0	2.5	1.1–2.3	0.9–1.2	2.2–2.5
GLU	mg/dl	Spring	Adult	175	110	27	109	51	205	58–171	51–68	160–205
			Denning^a^	48	131	14	127	108	161	107–164	104–111	155–173
			Subadult	41	111	29	108	59	184	59–184	59–74	155–184
		Fall	Adult	108	117	25	116	31	201	56–175	31–81	154–201
			Subadult	38	111	26	110	64	160	57–164	46–71	151–176
PHOS	mg/dl	Spring	Adult^a^	180	5.3	1.3	5.2	2.4	9.8	3–8.3	2.4–3.3	7.6–9.8
			Denning^a,c^	49	5.8	1.1	5.8	3.3	8.3	3.4–8.2	3.3–4.1	7.5–8.3
			Subadult^c^	42	6.0	1.5	5.9	3.5	10.1	3.6–10	3.6–4.1	8.4–10.1
		Fall	Adult	111	5.2	1.5	5.0	2.5	10.6	3–9.9	2.5–3.3	7.8–10.6
			Subadult	37	5.2	1.6	4.9	2.6	9.0	2.7–9.1	2.3–3.1	7.9–10.4
POT	mmol/l	Spring	Adult	176	4.4	0.4	4.4	3.0	5.5	3.6–5.2	3–3.8	5.1–5.5
			Denning^a^	48	4.2	0.5	4.1	3.0	6.1	3.1–6.0	3–3.7	5–6.1
			Subadult	42	4.5	0.4	4.5	3.7	5.3	3.7–5.2	3.7–4	5–5.3
		Fall	Adult	107	4.8	0.7	4.7	2.9	8.1	3.5–7.2	2.9–3.9	5.9–8.1
			Subadult	38	4.8	0.4	4.8	4.2	5.9	4.1–5.8	4–4.2	5.5–6.2
NA	mmol/l	Spring	Adult	178	137	4	137	124	154	128–148	124–130	144–154
			Denning	44	136	4	137	129	145	129–145	129–130	141–145
			Subadult	40	140	2	139	136	146	136–146	136–137	143–146
		Fall	Adult	112	143	10	142	112	180	123–168	112–130	162–180
			Subadult	38	143	6	141	133	159	133–159	na	na
TBIL	g/dl	Spring	Adult	181	0.3	0.1	0.3	0.1	0.7	0.2–0.5	0.1–0.2	0.5–0.7
			Denning	49	0.3	0.1	0.3	0.2	0.5	0.2–0.5	na	0.4–0.5
			Subadult	41	0.3	0.05	0.3	0.2	0.3	0.2–0.3	na	na
		Fall	Adult	108	0.3	0.1	0.3	0.2	0.7	0.2–0.6	na	0.5–0.7
			Subadult	38	0.3	0.1	0.3	0.2	0.5	0.2–0.5	na	na
TP	g/dl	Spring	Adult^a^	180	7.0	0.4	6.9	5.4	8.4	6–7.9	5.4–6.4	7.6–8.4
			Denning^c^	46	6.6	0.4	6.6	5.6	7.4	5.7–7.4	5.6–6	7–7.5
			Subadult^a,c^	41	6.8	0.2	6.8	6.4	7.3	6.5–7.3	6.5–6.6	7.2–7.3
		Fall	Adult	111	7.6	0.9	7.5	5.6	10.4	5.8–9.8	5.6–6.3	9.2–10.4
			Subadult	37	7.3	0.5	7.2	6.4	8.2	6.3–8.2	6.1–6.5	8–8.4

* ^*^Non-denning females are defined as ‘adult’.

a ^a^Subgroups with different letters are within the same season are significantly different (*P* < 0.01).

**Table 4:** RIs for male polar bears from the SB

Analytes	Units	Season	Subgroup	*n*	Mean	Standard deviation	Median	Minimum	Maximum	RI	90% Confidence interval for lower limit	90% Confidence interval for upper limit
ALT	U/l	Spring	Adult	162	50	25	43	11	160	16–126	11–25	96–160
			Subadult^a^	32	33	9	35	13	55	14–53	9–18	48–58
		Fall	Adult^*^	33	27	9	25	14	48	13–49	11–15	42–58
ALB	g/dl	Spring	Adult	162	5.5	0.4	5.5	3.8	6.4	4.7–6.3	3.8–4.8	6.1–6.4
			Subadult	31	5.5	0.3	5.4	4.9	6.2	4.9–6.2	4.7–5	6–6.3
		Fall	Adult	32	5.5	0.3	5.5	4.8	6.4	4.9–6.2	4.7–5	6–6.4
ALP	U/l	Spring	Adult	143	35.7	19.2	30.5	8.3	88.5	10.3–86.7	8.3–13	76.5–88.5
			Subadult^a^	32	68.7	43.3	48.8	11.5	166.5	12–167	na	na
		Fall	Adult	32	59.2	29.6	53.3	14.0	123.7	14–123.7	na	na
BUN	mg/dl	Spring	Adult	162	11.3	10.4	7.8	1.0	65.0	1–39.4	1–2	30–65
			Subadult	31	14.5	10.2	11.0	1.0	38.7	1–38.7	0.4–3	33.1–62
		Fall	Adult	32	17.6	12.4	13.0	3.0	49.0	2.6–53.1	2.3–3.9	39–66.6
CA	mg/dl	Spring	Adult	161	9.7	0.5	9.8	7.3	10.9	8.2–10.6	7.4–8.9	10.4–10.9
			Subadult^a^	31	10.1	0.5	10.1	9.4	11.1	9.2–11.2	8.9–9.4	10.9–11.4
		Fall	Adult	30	10.3	0.5	10.2	9.3	11.1	9.3–11.3	9.1–9.5	11–11.5
CREA	mg/dl	Spring	Adult	162	1.3	0.3	1.3	0.5	2.3	0.7–1.9	0.5–0.8	1.8–2.3
			Subadult^a^	32	1.0	0.2	0.9	0.4	1.5	0.5–1.4	0.4–0.6	1.3–1.5
		Fall	Adult	32	1.0	0.3	0.9	0.5	1.9	0.3–1.7	0.2–0.5	1.5–1.8
GLOB	g/dl	Spring	Adult	162	1.7	0.5	1.7	0.8	3.0	1–2.7	0.8–1.1	2.5–3
			Subadult^a^	32	1.5	0.4	1.5	0.5	2.3	0.6–2.3	0.4–0.8	2.1–2.5
		Fall	Adult	33	1.8	0.5	1.8	1.1	3.0	0.9–2.8	0.7–1.1	2.6–3
GLU	mg/dl	Spring	Adult	161	125	25	124	59	199	80–177	59–86	167–199
			Subadult	31	123	26	124	72	188	69–178	56–82	164–192
		Fall	Adult	30	119	24	124	47	148	51–155	47–82	148–162
PHOS	mg/dl	Spring	Adult	162	6.1	1.2	6.2	3.1	9.4	3.7–8.6	3.1–4.1	8.2–9.4
			Subadult	28	6.3	0.8	6.3	5.1	7.9	4.8–7.9	4.4–5.2	7.5–8.3
		Fall	Adult	33	5.8	1.6	5.5	3.4	9.1	3.2–9.6	2.8–3.7	8.5–10.8
POT	mmol/l	Spring	Adult	158	4.6	0.3	4.6	3.8	5.4	4–5.2	3.9–4.1	5.1–5.4
			Subadult	31	4.5	0.4	4.6	3.6	5.3	3.7–5.3	3.5–3.9	5.1–5.5
		Fall	Adult	33	4.6	0.5	4.6	3.4	5.5	3.6–5.5	3.4–3.9	5.3–5.7
NA	mmol/l	Spring	Adult	162	139	4	139	119	155	132–150	119–133	145–155
			Subadult	31	139	3	139	133	144	133–145	132–135	143–146
		Fall	Adult	32	140	3	140	133	146	133–146	132–135	144–148
TBIL	g/dl	Spring	Adult	161	0.3	0.1	0.3	0.1	0.4	0.2–0.4	0.1–0.2	0.4–0.4
			Subadult	32	0.3	0.1	0.3	0.2	0.4	0.2–0.5	na	na
		Fall	Adult	33	0.3	0.1	0.3	0.2	0.6	na	na	na
TP	g/dL	Spring	Adult	162	7.2	0.4	7.2	5.5	8.3	6.5–7.9	5.5–6.7	7.8–8.3
			Subadult^a^	30	7.0	0.3	7.1	6.3	7.8	6.3–7.8	6.2–6.5	7.6–8
		Fall	Adult	33	7.3	0.6	7.3	6.3	8.9	6.1–8.7	5.9–6.4	8.3–9

a ^a^Subgroups within the same season are significantly different (*P* < 0.01).

* ^*^Reference intervals for male polar bears in Fall include both adults and subadults.

### Spring RIs

We partitioned females captured in spring into three subgroups: non-denning adults, denning adults and subadults, consistent with expectations based on behaviour and physiology. Mean ALP activities of subadult females were nearly twice that of adult female polar bears and significantly different from both denning and non-denning adult females (Table 3; *Z*_subadult/adult_ = 5.64, *P* < 0.001, *Z*_subadult/denning_ = 6.24, *P* < 0.001). Denning females had significantly lower mean concentrations of ALB, POT and TP and mean ALT activity than non-denning adult females and significantly higher mean concentrations of GLU and CREA (Table 3).

Physiologic differences between females in spring based on the lower and upper confidence intervals of RI suggest limited differences in ALB levels, with denning females having a lower clinical decision interval (Friedrichs *et al.*, 2012). Similarly, denning females had higher minimum GLU values than non-denning adults and subadult females in spring. GLOB levels showed physiologic difference within female bears based on age and denning status, with denning females having lower GLOB levels than both non-denning adults and subadults.

Males sampled in spring were partitioned into adult and subadult age classes. The means of ALT, ALP, CA, CREA and GLOB were significantly different between the two age classes (Table 4). Similar to subadult females in spring, subadult males had significantly higher ALP activities, with an upper reference limit for subadults of 167 U/l, while the upper reference limit for adults was 89 U/l (*t* = 6.80, *P* < 0.001). ALT activities in subadult males ($\overline{x}$ = 33.27 U/l) were significantly lower than in adult males ($\overline{x}$ = 50.28 U/l, *t* = −3.69, *P* < 0.01). For each of these enzymes, the upper limit of the confidence intervals suggests a potential physiologic difference between the age classes, with increased ALP activity in subadults compared to adults, and the inverse relationship with ALT, with decreased activity in subadults compared to adults.

### Fall RIs

Fall sample sizes were smaller than spring sample sizes but still provided adequate numbers to calculate RI using an iterative (robust) statistical approach (Friedrichs *et al.*, 2012). Females were grouped into adults and subadults (Table 3). Differences in ALP between age classes were consistent across seasons, with subadult females having significantly higher mean ALP activities ($\overline{x}$ = 64.02 U/l) than adult females ($\overline{x}$ = 31.65 U/l, *t* = 5.69, *P* <  0.001). The higher upper confidence limit of ALP activity suggests a physiologic difference between the two age groups in fall. GLOB concentrations were the only other analyte where the mean differed significantly between subadult and adult females in fall (*z* = −2.68, *P* = 0.018). The upper limit of GLOB concentration in adult females suggests a physiologic difference between the two age classes. A single RI for each analyte for males in fall is reported in Table 4.

### Seasonal differences in RIs

**Figure 2:**
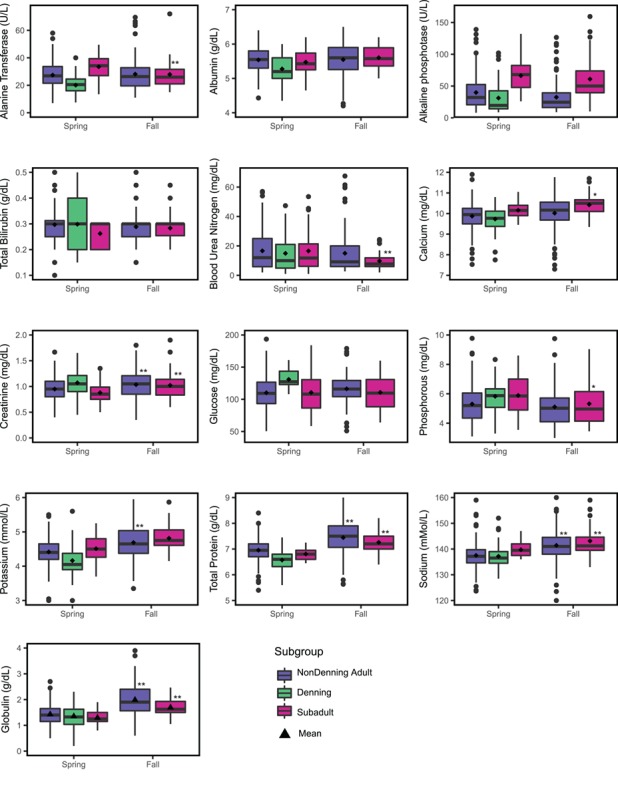
Seasonal differences of biochemical analytes for female polar bears with significance between like subgroups reported as ^*^P < 0.05, ^**^P < 0.01.

Adult females were separated into non-denning and denning females in the spring and combined in the fall (Fig. 2). We found significant differences in seasonal means for CREA ($\overline{x}$_fall_ = 1.04 mg/dl, $\overline{x}$_spring_ = 0.95 mg/dl; *t* = −2.96, *P* ≤ 0.01), POT ($\overline{x}$_fall_ = 4.75 mmol/l, $\overline{x}$_spring_ = 4.42 mmol/l; *t* = −5.01, *P* ≤ 0.001), TP ($\overline{x}$_fall_ = 7.57 g/dl, $\overline{x}$_spring_ = 6.96 g/dl; *t* = −7.45, *P* ≤ 0.001), NA ($\overline{x}$_fall_ = 142.79 mmol/l, $\overline{x}$_spring_ = 137.53 mmol/l; *t* = −6.23, *P* ≤ 0.001) and GLOB ($\overline{x}$_fall_ = 2.01 g/dl, $\overline{x}$_spring_ = 1.43 g/dl; *t* = −9.15, *P* ≤ 0.001) in adult females. Mean seasonal differences of analytes in subadult females were often statistically significant but minimal in magnitude, with the exception of BUN concentrations, suggesting limited seasonal differences on physiologic function. Subadult females showed greater seasonal variation with significant differences between seasonal means for BUN ($\overline{x}$_fall_ = 9.78 mg/dl, $\overline{x}$_spring_ = 16.58 mg/dl; *t* = 2.60, *P* ≤ 0.01), CREA ($\overline{x}$_fall_ = 1.02 mg/dl, $\overline{x}$_spring_ = 0.88 mg/dl; *t* = −2.70, *P* ≤ 0.01), POT ($\overline{x}$_fall_ = 4.81 mmol/l, $\overline{x}$_spring_ = 4.51 mmol/l; *t* = −3.44, *P* ≤ 0.001), NA ($\overline{x}$_fall_ = 143.14 mmol/l, $\overline{x}$_spring_ = 139.73 mmol/l; *t* = −3.38, *P* ≤ 0.01), TP ($\overline{x}$_fall_ = 7.26 g/dl, $\overline{x}$_spring_ = 6.81 g/dl; *t* = −5.72, *P* ≤ 0.001) and GLOB ($\overline{x}$_fall_ = 1.69 g/dl, $\overline{x}$_spring_ = 1.29 g/dl; *t* = −6.01, *P* ≤ 0.001). Of these statistically different analytes, only GLOB concentration suggests a physiologic difference between spring and fall in both adults and subadults. BUN in subadult females was the only analyte to significantly increase in fall, all other significantly different analytes showed decreased activity and concentration in spring regardless of age.

**Figure 3:**
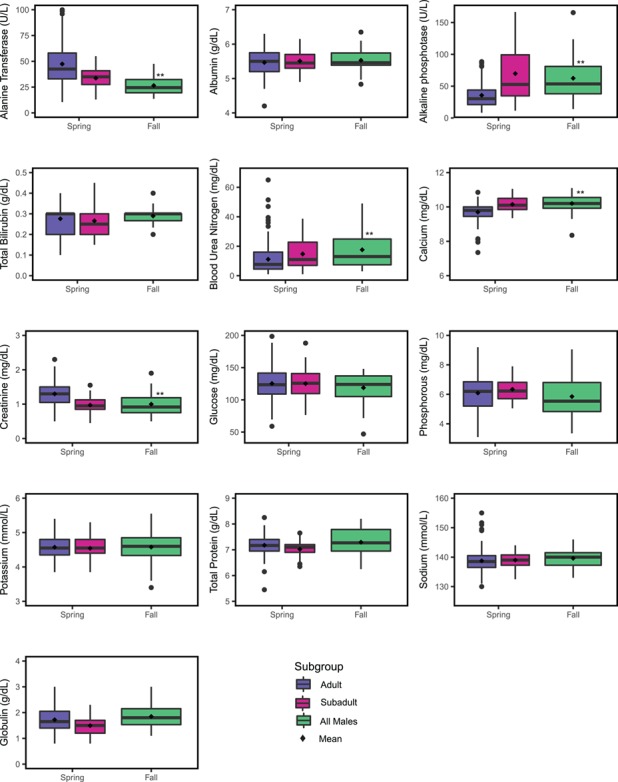
Seasonal differences of biochemical analytes for male polar bears with significance between like subgroups reported ^**^P < 0.001

Males were separated into subadults and adults in the spring and combined in the fall (Fig. 3). For adult males, ALP was greater in fall ($\overline{x}$ = 59.17 U/l) than spring ($\overline{x}$ = 35.61 U/l, *t* = −3.69, *P* < 0.01), as was CA ($\overline{x}$_fall_ = 10.26 mg/dl, $\overline{x}$_spring_ = 9.70 mg/dl; *t* = −4.16, *P* ≤ 0.001) and BUN ($\overline{x}$_fall_ = 17.58 mg/dl, $\overline{x}$_spring_ = 11.70 mg/dl; *t* = −2.91, *P* ≤ 0.01). In each of these cases, the RI shifted to the right in fall, suggesting a physiologic difference. ALT also showed a significant difference between spring and fall ($\overline{x}$_fall_ = 26.51 U/l, $\overline{x}$_spring_ = 47.87 U/l; *t* = 5.10, *P* ≤ 0.001) with the upper limit of ALT activity in spring being more than twice as high as fall activity (Fig. 3). Mean CREA concentration was significantly lower in fall ($\overline{x}$_fall_ = 1.00 mg/dl, $\overline{x}$_spring_ = 1.24 mg/dl; *t* = 4.09, *P* ≤ 0.001); however, the physiologic importance of this difference is likely minimal.

## Discussion

Although previous research has reported blood analyte values for polar bears, these reports have examined fewer analytes and smaller numbers of bears (e.g. Lee *et al.*, 1977; Nelson *et al.*, 1983; Derocher *et al.*, 1990; Ramsay *et al.*, 1991; Tryland *et al.*, 2002; Rode *et al.*, 2014; Whiteman *et al.*, 2017, 2018). Our goal was to utilize a large data set to create robust RI based on a well-studied subpopulation that can serve as a foundation for relating biochemical analytes and polar bear health in this and other subpopulations.

Assessment of health in reference subjects is of paramount importance is establishing RI and yet is challenging in free-living wildlife owing to a single point-in-time examination. Inclusion of unhealthy subjects has the potential to widen the RI, rendering it less sensitive for detecting deviation from healthy analyte distributions (Johansen and Christensen, 2018). In order to minimize inclusion of potentially unhealthy subjects, specific criteria were defined in order to exclude potentially unhealthy subjects (see Materials and methods). Our examination and exclusion of outliers from the reference population warranted our inclusion of samples from the last four decades despite accelerating rates of environmental change and habitat perturbation in the Arctic (Harr *et al.*, 2018).

Our results were consistent with related work on large carnivores that found higher ALP activity in subadult/juveniles than adults: wolves (*Canis lupus*, Thoresen *et al.*, 2009), grizzly bears (*Ursus arctos horribilis*, Brannon, 1985) and polar bears (Lee *et al.*, 1977; Tryland *et al.*, 2002). ALP is an enzyme in both liver and bone and is involved in bone growth and remodelling. ALP is thus expected to be higher in subadults regardless of season. BUN concentrations were lowest in denning females, which likely reflects extended fasting and the energetic demands of raising young. Females with cubs of the year are often captured shortly after leaving the den, leaving little time for hunting prior to capture (Derocher *et al.*, 1990). As access to food in spring increases, we would expect BUN concentrations to increase. Denning females also had significantly higher GLU concentrations than both adults and subadult females in spring. This difference may be related to increased GLU requirements during lactation (Bell and Bauman, 1997). These results are inconsistent with the findings of Halloran and Pearson (1972) and Matula *et al.* (1980) in brown and black (*Ursus americanus*) bears, respectively, but both authors note inconsistencies among published reports relating blood GLU concentration to denning and lactation (e.g. Lee *et al.*, 1977; Franzmann and Schwartz, 1988; Stenvinkel *et al.*, 2013).

Seasonal differences in analytes are likely a response to changes in nutrition and behaviour. In the western Hudson Bay subpopulation, polar bears are forced on shore when the sea ice melts in summer and have little access to food until the ice re-forms in the fall (Atkinson and Ramsay, 1995). Ramsay *et al.* (1991) reported a pronounced seasonal variation in BUN concentrations for western Hudson Bay bears, which averaged 48.4 ± 1.8 mg/ml for individuals captured on sea ice in spring and 19.1 ± 5.4 mg/ml for those captured on land in summer. In the SB, season-specific BUN and CREA RI were lower than those reported elsewhere (Nelson *et al.*, 1983; Ramsay *et al.*, 1991; Tryland *et al.*, 2002). Our BUN RI for adult females in spring was 2.4–48.80 mg/dl with a mean of 16.7 mg/dl. Thus, our maximum spring value equaled the mean spring value reported for western Hudson Bay, while our mean spring value matched that reported for western Hudson Bay bears in the summer that had been fasting on land. Similarly, spring and fall CREA RIs from our study were substantially lower than spring and summer CREA ranges and RI previously reported for the western Hudson Bay and Barents Sea subpopulations (Nelson *et al.*, 1983; Ramsay *et al.* 1991; Tryland *et al.*, 2002). Explanations for these differences between the SB and other subpopulations could be due to disparate ice conditions during the respective study periods (Stroeve *et al.*, 2012) or to differences in biological productivity between subpopulations (Rode *et al.*, 2018).

Many researchers have used BUN and CREA to assess fasting in polar bears. Recently, Rode *et al.* (2018) documented declines in the ratio of BUN to CREA, which is an index of feeding over the previous 7 days and found increased rates of fasting in SB polar bears between 1983 and 1999 and 2000 and 2016. Pagano *et al.* (2018) and Whiteman (2018) supported this finding noting increases in metabolic rates due to increased energy expenditure and declines in hunting opportunities related to deteriorating sea ice habitat. While not the goal of this research, our work provides a basis from which to continue investigations into physiologic adjustments resulting from a changing climate. Using deviations from RI, we can better understand how abiotic and biotic conditions such as changes in sea ice are impacting polar bears and determine the best metrics for surveillance and monitoring.

Our work adds to the understanding of the blood biochemistry of polar bears. Our large sample size permitted biologically appropriate subgrouping, allowing us to examine differences in age class and reproductive status, the classifications used for managing polar bear populations. Nevertheless, our study has certain inherent limitations. For example, the declining availability of sea ice in the SB during summer and fall precluded the continuation of safe captures limiting our ability to calculate summer RI that included data beyond 2009. We caution that although we report a number of statistical differences for analytes across subgroups and between seasons, it is important to consider the functional importance of these differences. For example, mean TP levels showed significant differences between all subgroups for females in spring. However, the calculated values suggest minimal influence on physiologic function and critical decision limits. To clarify the functional significance of the differences we have documented, it would be useful to determine how the analytes we measured vary with known disease states. To inform relationships between disease and blood biochemistry, we suggest examining zoo-managed polar bears as well as wild polar bears with known pathological conditions (Atwood *et al.*, 2015) to establish critical values for these physiologic markers.

We also acknowledge that RI created for one subpopulation using one analytical system may not reflect the variability of values observed in other subpopulations or by other methods. However, we provide a well-documented and robust resource for comparisons within and across the circumpolar population of polar bears. Our work is therefore most relevant to monitoring the SB subpopulation of polar bears, including detecting changes in physiologic function that may reflect subclinical and clinical disease in individuals and populations. In general, RIs provide a baseline for assessing health, and deviation from these RIs may signal an adaptive physiologic response. The SB subpopulation of polar bears is one of the most well studied; therefore, associations between stressors and physiologic responses documented for the SB subpopulation can be used to inform monitoring and management decisions both for this population and potentially for other subpopulations even with different baseline values. Furthermore, combining baseline physiologic data such as ours with complementary data on hematology (Kirk *et al.*, 2010) and transcriptomics (Bowen *et al.*, 2015, 2015b), as well as data on diet and nutrition (McKinney *et al.*, 2017), reproduction (Rode *et al.*, 2010), behaviour (Whiteman *et al.*, 2015; Atwood *et al.*, 2016; Lillie *et al.*, 2018; Pagano *et al.*, 2018) and pathogen exposure (Atwood *et al.*, 2015, 2017) could help identify how polar bears might react and adapt to external stressors such as infectious diseases, environmental catastrophes and climate change (Stroeve *et al.*, 2012; Laidre *et al.*, 2015). This set of RI for SB polar bears provides a robust foundation necessary to make temporal and spatial observations on the overall health of polar bears as well as comparisons both within and among subpopulations facing myriad ecological challenges.
